# 3D segmentation of dental crown for volumetric age estimation with CBCT imaging

**DOI:** 10.1007/s00414-022-02898-8

**Published:** 2022-10-05

**Authors:** Rizky Merdietio Boedi, Simon Shepherd, Fahmi Oscandar, Scheila Mânica, Ademir Franco

**Affiliations:** 1 Centre of Forensic and Legal Medicine and Dentistry, University of Dundee, Dundee, UK; 2 Department of Dentistry, Faculty of Medicine, Universitas Diponegoro, Semarang, Indonesia; 3 Department of Oral Surgery, School of Dentistry, University of Dundee, Dundee, UK; 4 Departement of Oral and Maxillofacial Radiology - Forensic Odontology, Faculty of Dentistry, Universitas Padjadjaran, Bandung, Indonesia; 5 Division of Forensic Dentistry, Faculdade São Leopoldo Mandic, Campinas, Brazil

**Keywords:** Cone-beam computed tomography, Dental age estimation, Forensic dentistry, Tooth attrition, Secondary dentine

## Abstract

In adult dental age estimation, segmentation of dental volumetric information from different tooth parts using cone-beam computed tomography (CBCT) has proven beneficial in improving the regression model reliability. This segmentation method can be expanded in the crown part since the volumetric information in the crown is affected by attrition in the enamel and secondary dentine in the dentine and pulp chamber. CBCT scans from 99 patients aged between 20 and 60 were collected retrospectively. A total of 80 eligible teeth for each tooth type were used in this study. The enamel to dentine volume ratio (EDVR), pulp to dentine volume ratio (PDVR) and sex were used as independent variables to predict chronological age (CA). The EDVR was not affected by PDVR. The highest *R*^*2*^ was calculated from the maxillary canine (*R*^*2*^ = 0.6). The current approach in crown segmentation has proven to improve model performance in anterior maxillary teeth.

## Introduction

Since the conception of Gustafson’s method in 1950 about morphological dental age estimation in adults [1], much research has been carried out to investigate tooth regressive changes in a less invasive way. For instance, research in the 1990s enabled the visualization of regressive changes in the pulpal space related to secondary dentine deposition [2]. These improvements contributed to the forensic field with dental age estimation tools applicable to the living or without damaging the teeth of the deceased [3, 4]. One of the technical counterpoints of the imaging tools is the difficulty of including all the original age-related parameters advocated by Gustafson. On the one hand, secondary dentine deposition, attrition, and root resorption can be assessed with radiographical imaging; on the other hand, root translucency, cementum apposition, and periodontosis are not observable.

Although the predictor variable used to estimate the chronological age (CA) in radiographs is limited, improvements in image acquisition technology can directly help forensic odontologists observe various dental morphological changes [5–8]. Furthermore, with the introduction of three-dimensional imaging such as cone-beam computed tomography (CBCT), accurate quantification of the tooth was possible through volumetric approximation using voxel counting software [9].

In CBCT, the current state-of-the-art methods rely primarily on pulp-to-tooth volume ratios [10]. These methods consider the regressive changes in tooth morphology as an entire unit and preclude specific analysis on how individual tooth components correlate to CA. Because attrition is restricted to the crown and resorption is restricted to the root, tooth-part segmentation is justified to understand the effects of regressive morphological changes and their correlation with the chronological age separately per tooth part [11]. A recent study endorsed positive outcomes using the segmentation of dental volume for the crown and root parts [12]. A deeper look, however, could be dedicated specifically to the crown part since three dental morphological changes can be observed in this region: (1) the reduction of enamel due to attrition, (2A) the increasing volume of dentine, and (2B) the reduction of pulp chamber due to secondary dentine deposition [13]. Therefore, to further investigate the relationship between different crown morphological changes and the CA, this study aimed to assess the correlation between CA and the different volumetric information quantified from the crown, namely enamel volume (EV), dentine volume (DV), and pulp chamber volume (PCV).

## Material and methods

This observational analytical cross-sectional study was approved by the Research Ethics Committee of Universitas Padjajaran No. 899/UN6.KEP/EC/2021. CBCT scans from 99 patients with a mean age of 40.69 ± 11.24 were retrospectively collected in this study. The sample included 45 males and 54 females from Bandung, Indonesian origin, with CA cohorts ranging from 20 to 59.99 years old. The CA was calculated from the difference between the scan date and the date of birth.

CBCT scans were taken in Universitas Padjajaran Dental Hospital using Instrumentarium Dental OP300 (Instrumentarium Dental, Tuusula, Finland) with patient-specific exposure settings. All CBCT scans used in this study were taken for various diagnostic and therapeutic purposes, and no patient was exposed to radiation for the sole purpose of research.

Sound maxillary anterior teeth, namely canine (C), lateral incisor (Li), and central incisor (Ci), were used in this study with the inclusion criteria of fully erupted, closed apex, and visible cementoenamel junction (CEJ). Teeth with restorations, impaction, lesions, and accessory canal were excluded. From the 99 selected CBCT scans, 80 teeth were selected for each tooth type. The minimum sample used in this study was calculated using a priori power analysis using 3 predictors, 0.3 effect size, and 0.95 power resulting in a minimum sample of 62 per tooth [14]. The total tooth sample used in this study is presented in Table 1.

**Table 1:** The number of examined teeth classified by the tooth type for each sex

Group	Age (years)	M			F		
		C	Li	Ci	C	Li	Ci
1	20–24.99	5	5	5	5	5	5
2	25–24.99	5	5	5	5	5	5
3	30–34.99	5	5	5	5	5	5
4	35–35.99	5	5	5	5	5	5
5	40–44.99	5	5	5	5	5	5
6	45–49.99	5	5	5	5	5	5
7	50–54.99	5	5	5	5	5	5
8	55–59.99	5	5	5	5	5	5

^*C*^ canine, *Li* lateral incisor, *C*, central incisor

The crown segmentation method starts by importing the CBCT in Digital Imaging and Communications in Medicine (DICOM) format to ITK-SNAP (version 3.8, ITK-SNAP, UPenn & UNC, USA). Segmentation aims to observe and create three measured parameters, namely PCV, DV, and EV, in this specific order using the built-in region of interest (ROI) function. The crown segmentation started by placing the ROI in the sagittal view’s highest or most apical CEJ portion.

**Fig. 1:**
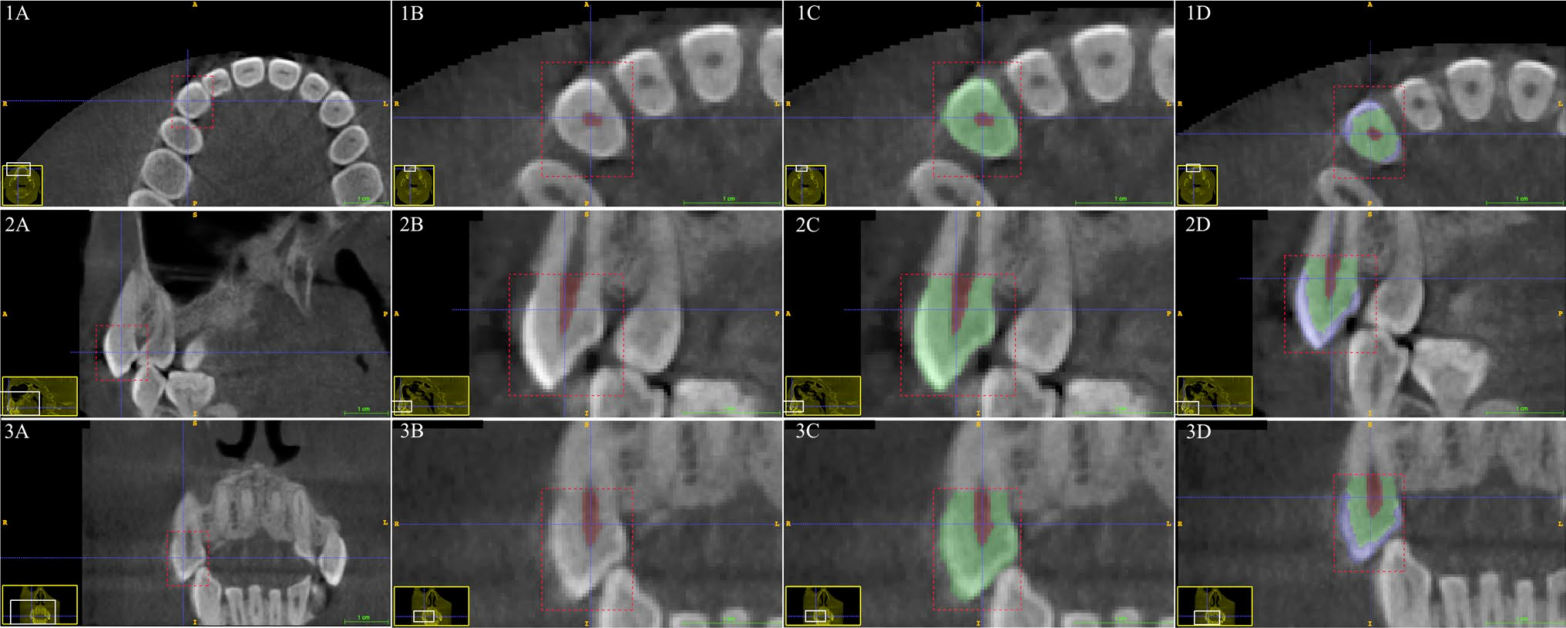
Segmentation sequence in the ITK-SNAP interface for the maxillary canine. Segmentation sequence as seen in column 1 (A–D) = axial view, column 2 (A–D) = sagittal view, column 3 (A–D) = coronal view. Row A = initial region of interest placement (red box), row B = pulp chamber volume label (red), row C = whole crown segmentation (green), row D = separation between dentine volume (green) and enamel volume (blue). The yellow window (bottom left) visualizes the current navigation region from the overall CBCT scan section plane with white inset box for the enlarged image. The blue cross represents the target point of interest for CBCT scan navigation

**Fig. 2:**
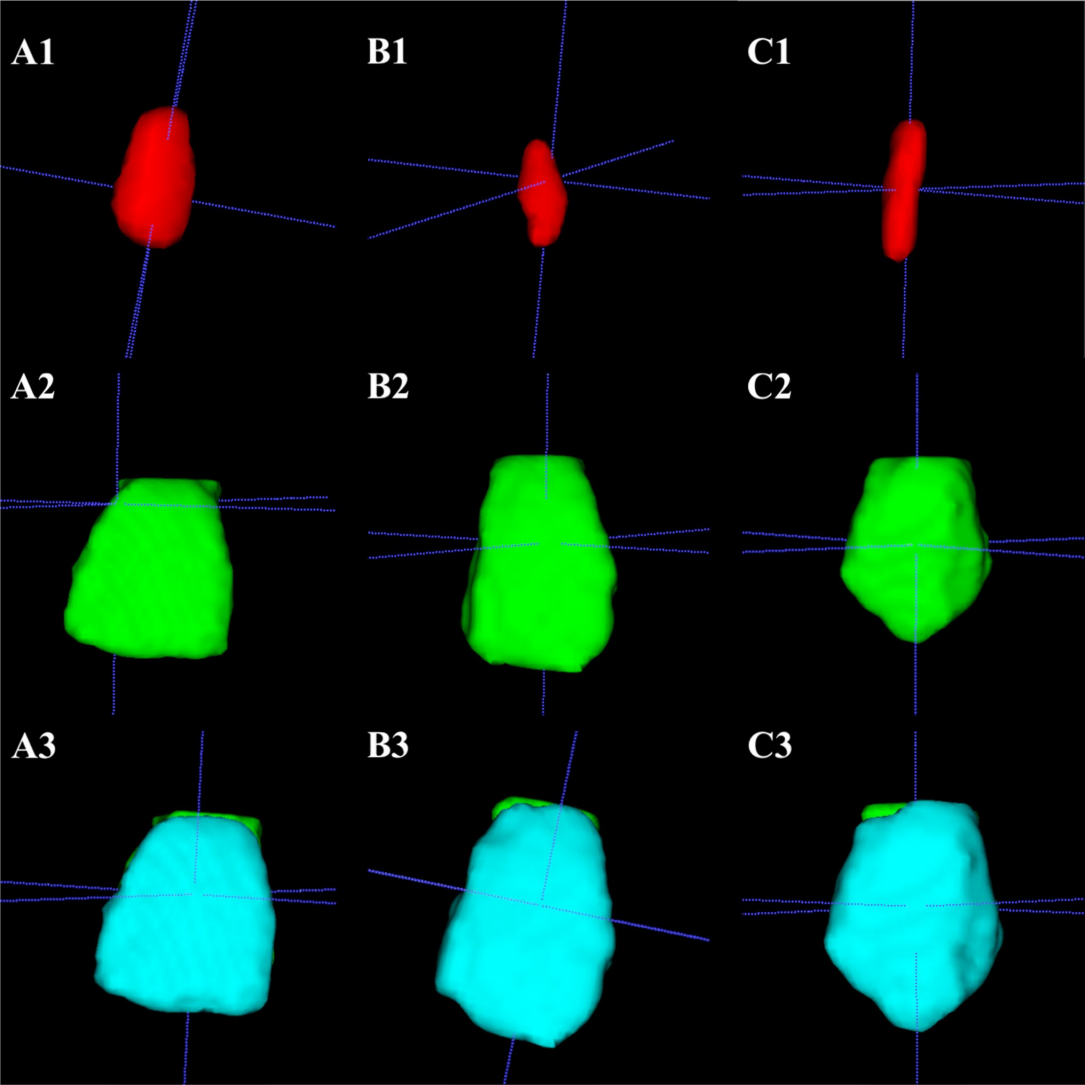
Three-dimensional view of maxillary anterior tooth segmentation sequence. A = central incisor, B = lateral incisor, C = canine, 1 = pulp chamber volume, 2 = whole crown volume before enamel segmentation, 3 = crown volume separation between dentine (green) and enamel (blue) volume

First, PCV segmentation was accomplished by selecting a “label” to paint above the “clear label”. Then, the contrast thresholding in the “pre segmentation mode” was set to a minimum in the lower and upper thresholds as desired to segment the PCV fully. Second, DV segmentation was performed with the same method, with the difference in the contrast thresholding by setting the minimum value to the upper and lower threshold as desired to segment the whole crown without differentiating between dentine and enamel. Finally, the EV segmentation started by selecting a label to paint over the chosen label for DV (i.e. if the DV label was set to label 2, then the paint over function was set to label 2). Contrast thresholding for EV should only select the enamel layer in the scan. Any measured parameter area that was not covered by the label or any leaking label to an adjacent structure can be added or deleted manually using the brush function (Figs. 1, 2).

To ensure the method’s reproducibility, the intraclass correlation coefficient (ICC) for the intra- and inter-observer agreement was calculated using 20 randomly selected teeth with 2-week intervals between observations 1 and 2. The first observer (RMB) is a forensic odontologist with experience in dental age estimation. The second observer (KA) is an oral radiologist recruited for dental age estimation research.

The quantified volumetric measurements were then recorded in Microsoft Excel 365 (Microsoft, Redmond, USA) and calculated as a ratio parameter, namely pulp to dentine volume ratio (PDVR = $\frac{PCV}{DV}$*)* and enamel to dentine volume ratio (EDVR = $\frac{EV}{DV}$). Data analysis and visualization were done using R (version 3.4.0, R Foundation for Statistical Computing, Vienna, Austria). The relationship between ratio parameters and CA was quantified using Pearson correlation coefficient (*r*) and visualized with Locally Weighted Scatterplot Smoothing (LOWESS). Furthermore, a regression model was created with CA as the dependent variable, ratio parameters as the predictor, and gender as a covariate. The regression model was built on caret using fivefold cross-validation and two repetitions to protect the model from overfitting [15]. Randomization inside R was controlled by setting the seed parameter to 29 before running each model (set.seed = 29). The model’s performance evaluation was done by calculating the coefficient of determination (*R*^*2*^), root mean squared error (RMSE), and mean absolute error (MAE). To identify multicollinearity or linear dependencies between predictor variables, variance inflation factor (VIF) analysis was calculated with a cut-off value of 5 to indicate the presence of multicollinearity [16].

Additionally, we operated a quality-control correlation analysis to observe the influence of tooth angulation on the quantification of ratio parameters [12]. Forty-two randomly selected samples for each tooth type were measured. Tooth angulation was calculated using the angle tool using Fiji ImageJ (National Institutes of Health, Maryland, USA) in the sagittal view [17]. The horizontal line was drawn parallel to the image of the incisal tooth tip, and the vertical line was drawn parallel to the sagittal tooth section.

## Results

**Fig. 3:**
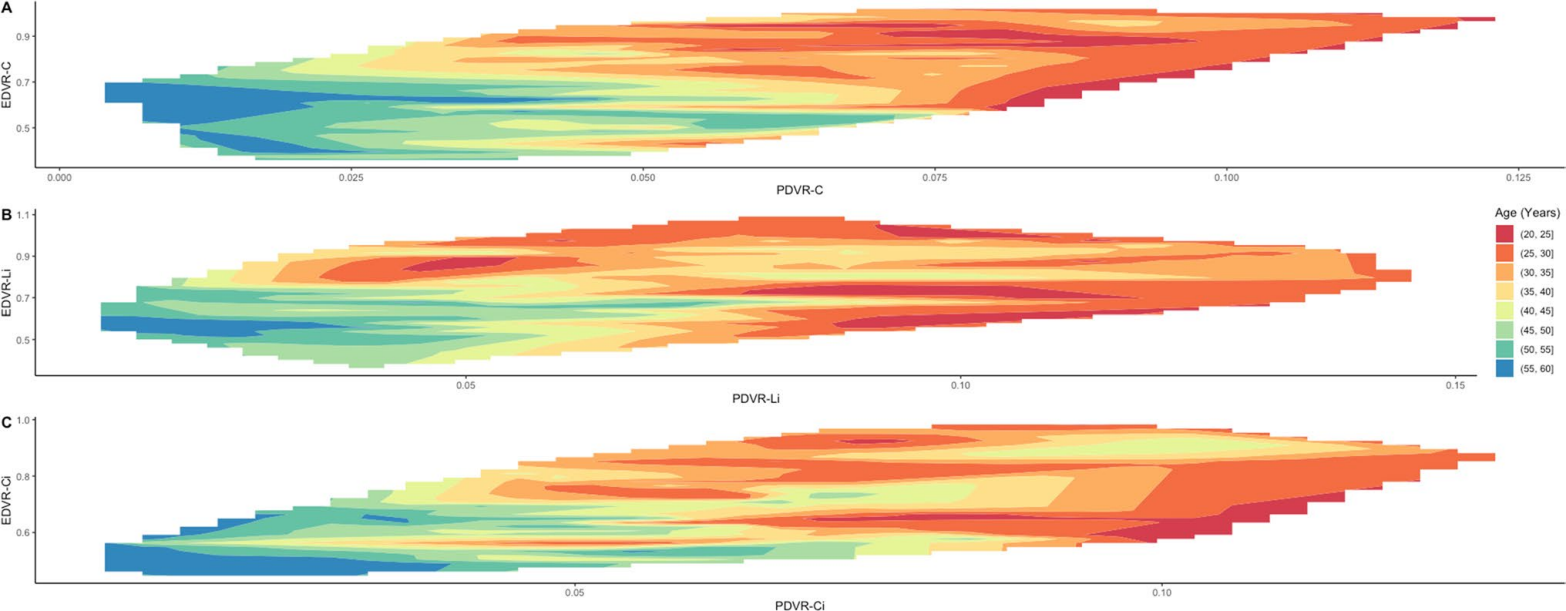
Heatmap graphic depicting a three-way relationship between chronological age, pulp to dentine volume ratio (PDVR) and enamel to dentine volume ratio (EDVR) in canine (A), lateral incisor (B), and central incisor (C). X-axis: pulp to dentine volume ratio. Y-axis: enamel to dentine volume ratio. Color grid shade on each coordinate in X and Y axis corresponds to age range from red (20–25 years old) to blue (55–60 years old)

**Fig. 4:**
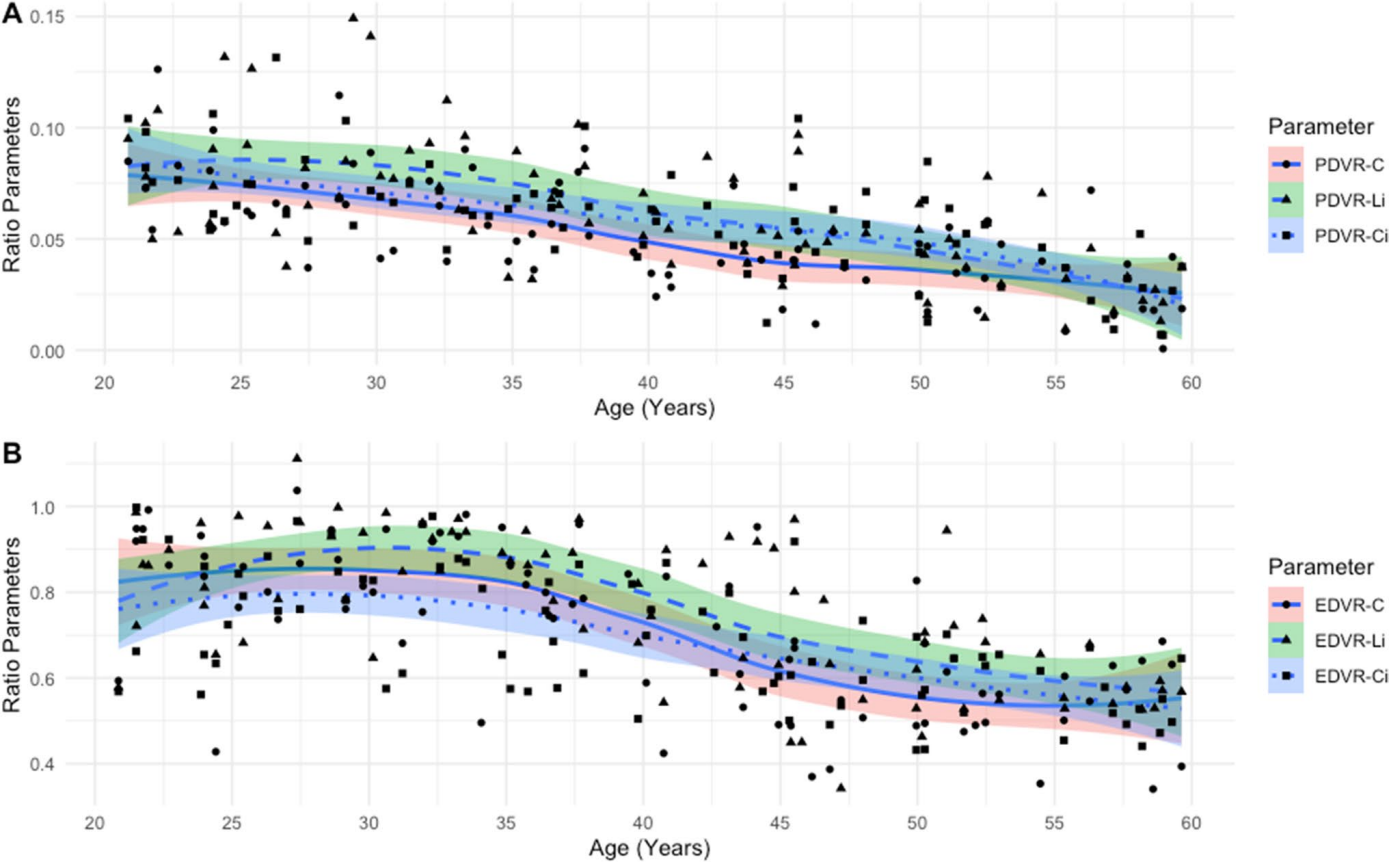
Decreasing ratio parameters (**A** = pulp to dentine volume ratio, **B** = enamel to dentine volume ratio) alongside chronological age depicted by the LOWESS line with linear (**A**) and non-linear (**B**) variations. C = canine, Li = lateral incisor, Ci = central incisor, PDVR = pulp to dentine volume ratio, EDVR = enamel to dentine volume ratio

The method reproducibility for intra-observer was 0.84, 0.91, and 0.88, and for inter-observer was 0.86, 0.7, and 0.67 for PCV, DV, and EV, respectively. Each segmentation took approximately 25 min for each tooth. A moderately strong relationship was found between ratio parameters and CA—confirming the regressive aspect of their morphological changes with age, and a fair relationship within the ratio parameters, all with significant results (*p* < 0.01, Table 2, Fig. 3) [18]. There was no correlation between tooth angulation and every ratio parameter (*p* > 0.05). The LOWESS line indicates a linear relationship between PDVR and CA and a non-linear relationship between EDVR and CA (Fig. 4).

**Table 2:** The correlation coefficient between ratio parameters and chronological age

Tooth		CA	PDVR	EDVR
C	CA	-	− 0.67	− 0.66
	PDVR	− 0.67	-	0.56
	EDVR	− 0.67	0.56	-
Li	CA	-	− 0.65	− 0.61
	PDVR	− 0.65	-	0.47
	EDVR	− 0.61	0.47	-
Ci	CA	-	− 0.68	− 0.61
	PDVR	− 0.66	-	0.56
	EDVR	− 0.61	0.56	-

^*C*^ canine, *Li* lateral incisor, *Ci* central incisor, *CA* chronological age, *PDVR* pulp to dentine volume ratio, *EDVR* enamel to dentine volume ratio

Regression models for each tooth were reported in Table 3, with no sex covariate being significant. The highest value of *R*^*2*^ from the C model (*R*^*2*^ = 0.6) and the lowest *R*^*2*^ from the Ci model (*R*^*2*^ = 0.53). Although there was a significant relationship within the ratio parameters (Table 2, *p* < 0.01), there was no multicollinearity detected in every model (VIF < 5). Also, adding a polynomial function to address the non-linearity between EDVR and CA was insignificant in every model.

**Table 3:** Regression model for each tooth type

Tooth	Regression model	*R*^*2*^	RMSE	MAE
C	$y =69.06-26.34 EDVR-205.06 PDVR$	0.6	7.71	6.11
Li	$y =76.05- 25.93 EDVR-176.43 PDVR$	0.55	7.94	6.45
Ci	$y =71.75-27.48 EDVR- 219.08 PDVR$	0.53	8.04	6.57

^*C*^ Canine, *Li* lateral incisor, *Ci* central incisor, *R2* coefficient of determination, *RMSE* root mean squared error (years), *MAE* mean absolute error (years)

## Discussion

The utilization of CBCT can improve the error rates for adult dental age estimation, especially with the currently available volumetric segmentation. However, the application of the methodology itself is challenging. Although the software utilized in this research (ITK-SNAP) is open-source, making the method more accessible, the whole process of crown segmentation still requires a steep learning curve. Phulari et al. (2021) had a similar observation regarding the evolution of adult dental age estimation. The authors explain that these methods are not universally used and that newer methods need first proper reproducibility to reach (later) better error rates [19].

In the present study, linear regression models utilizing three different morphological changes as ratios in anterior maxillary crowns led to *R*^*2*^ values that ranged from 0.53 to 0.6. Molina et al. (2021) reported a maximum *R*^*2*^ value of 0.377 using upper incisors by assessing the pulp to crown volume ratio [20], whilst Zhang et al. (2019) reported an *R*^*2*^ value of 0.419 by using enamel to pulp volume in impacted mandibular third molars [13]. Asif et al. (2018) reported an *R*^*2*^ value of 0.775 for the ratio between pulp to crown volume in maxillary central incisors of Malay adults [21]. The outcomes of the present study fall in between the values reported by the scientific literature in 2018 [18] and 2021 [17]. In other words, we found an overall moderate effect of both ratios assessed for adult age estimation in the present study. Differences between populations, however, may occur, justifying population-specific recalibration of the regression model to best fit the age estimation practice worldwide.

The current crown segmentation approach aims to address and quantify attrition through EV calculations. The role of attrition in adult dental age estimation has been disputed in the scientific literature [22]. For example, Xiaohu et al. (1992) advocate that a metric approach could give better estimates when compared to ordinal stages [23], whilst Li et al. (1995) and Ajmal et al. (2001) found otherwise [24, 25]. Still, the consensus relies on the combination of regressive parameters for a more comprehensive age estimation approach—since attrition solely can be affected by various external factors [23]. Therefore, our current approach to address this issue is combining the deposition of secondary dentine from DV and PCV with attrition from EV quantification. Additionally, predicting the CA by using linear regression is possible due to the (numerical) volumetric data given by the calculations, unlike the ordinal (i.e. staging) data which requires a Bayesian approach to achieve optimal results [26].

In this study, the sample criteria did not exclude occlusion status. Although previous studies reported that the effect of occlusion in anterior tooth attrition is indecisive [27], recent research by Liu et al. (2014) reported that the anterior maxillary tooth has an active role in both masticatory and excursive jaw movements, therefore giving a substantial loss of dentine and enamel on the incisal surfaces [28]. Other conditions in the occlusion may also affect the progress of attrition, such as overbite and anterior protected articulation [29, 30]. The effects of this condition on the dental age estimation results in the adult population are little known. Future research should evaluate the correlation between dental attrition and CA in other tooth regions, especially in the posterior tooth, as there is a significant difference between enamel reduction in functional and non-functional cusp [31].

This research found a significant correlation (*p* < 0.01) within the ratio parameters, namely the correlation between PDVR and EDVR. Although both variables had a significant correlation, there was no indication of multicollinearity in our model (VIF < 5). Nudel et al. (2021) presents a similar observation on the relationship between attrition and secondary dentine in premolars using Micro CT. They found that the formation of secondary dentine—which mainly affects the PDVR—is not affected by the attrition that affects EDVR [32]. Hence, separating both regressive morphological changes in the crown region may improve the model’s reliability without introducing collinearity.

Compared to a previous study using a similar population cohort, the crown segmentation increases the model performance from 0.03 to 0.22 in the *R*^*2*^ value [12]. Although this is a significant improvement, the time consumption to perform a single crown segmentation increases the labour time by approximately 20 min. Additionally, the method’s reproducibility had a mild reduction of ± 0.1 in the ICC analysis. Consequently, each forensic odontologist needs to consider the utilization of a methodology, as the risk (i.e. time consumption, method reproducibility) and reward (i.e. model performance, accessibility) may affect the optimal workflow of dental age estimation.

## Conclusion

The current approach to crown segmentation methodology by separating PCV, DV, and EV has proven useful in improving model performance in anterior maxillary teeth. However, we advise that the current method should be performed by trained experts, as there is a noticeable steep learning curve and prolonged time consumption in applying the 3D segmentation process. Furthermore, using the current model for external populations should be considered carefully since the current outcomes might be population specific.

## Data Availability

Data generated or analysed in this study are available from the corresponding author on reasonable request.
